# *Cryptococcus neoformans*: Diagnostic Dilemmas, Electron Microscopy and Capsular Variants

**DOI:** 10.3390/tropicalmed4010001

**Published:** 2018-12-20

**Authors:** Monica Birkhead, Serisha D. Naicker, Nozuko P. Blasich, Ivy Rukasha, Juno Thomas, Charlotte Sriruttan, Shareef Abrahams, Grisselda S. Mavuso, Nelesh P. Govender

**Affiliations:** 1 National Institute for Communicable Diseases—a Division of the National Health Laboratory Service, 1 Modderfontein Road, Sandringham 2192, South Africa; serishan@nicd.ac.za (S.D.N.); nozukob@nicd.ac.za (N.P.B.); ivyr@nicd.ac.za (I.R.); junot@nicd.ac.za (J.T.); CharlotteS@nicd.ac.za (C.S.); neleshg@nicd.ac.za (N.P.G.); 2 School of Pathology, Faculty of Health Sciences, University of the Witwatersrand, 7 York Road, Parktown, Johannesburg 2193, South Africa; 3 National Health Laboratory Service, Port Elizabeth Provincial Hospital, Cnr Buckingham & Eastbourne Road, Port Elizabeth 6001, South Africa; Shareef.Abrahams@nhls.ac.za; 4 National Health Laboratory Service, Tambo Memorial Hospital, Cnr Hospital & Railway Street, Boksburg 1459, South Africa; grisselda.mavuso@nhls.ac.za

**Keywords:** *Cryptococcus neoformans*, capsule, lateral flow assay, molecular characterisation, transmission electron microscopy

## Abstract

Two cases of cryptococcal meningitis went undetected by a cryptococcal antigen (CrAg) lateral flow assay on blood in a reflex CrAg screen-and-treat programme in South Africa, although *Cryptococcus neoformans* was identified by culturing the cerebrospinal fluid specimens. Further investigations into these discordant diagnostic results included multilocus sequence typing (which showed no mutations in the CAP59 gene) and transmission electron microscopy using a capsule-staining protocol (which revealed a >50% reduction in capsular material in both cases, relative to a control culture). A multi-disciplinary approach for resolving discordant diagnostic test results is recommended.

## 1. Introduction

In October 2016, South Africa (SA) implemented the national cryptococcal antigen (CrAg) reflex screening for persons living with HIV who have a CD4+ T-lymphocyte (CD4) count of <100 cells/µL [1]. A positive screening result prompts pre-emptive antifungal treatment for asymptomatic persons, or targeted treatment for symptomatic persons, with cryptococcal meningitis [2,3,4]. This cost-effective and globally-recommended strategy has been shown to reduce mortality from cryptococcal meningitis, which has a fatal outcome in more than half of cases in routine care in SA [5,6,7]. Screening is performed using a CrAg lateral flow assay (LFA), which can detect all *Cryptococcus neoformans* serotypes by immunochromatographic identification of the constituents of the cryptococcal polysaccharide capsule, of which the most abundant is glucuronoxylomannan [6,8,9]. A recent systematic review of the sensitivity and specificity of this assay on a variety of specimens (serum, plasma, whole blood, cerebrospinal fluid) gave median values of 100% and 97.7% respectively, versus the standard positive culture from cerebrospinal fluid (CSF) [6].

The National Institute for Communicable Diseases (NICD) provides technical support to all (approximately 50) public-sector CD4 laboratories participating in the national screening programme, and to diagnostic microbiology laboratories, by monitoring CrAg test discrepancies through inter-laboratory quality assessments and clinical referrals. We report on two such cases that were referred for further investigation.

## 2. Materials and Methods

The LFA that is used in the South African HIV screen-and-treat programme is the IMMY assay (Immuno-Mycologics, Norman, OK, USA). This was performed routinely at the CD4/microbiology laboratories in accordance with the manufacturer’s instructions, with additional testing of serial dilutions at the reference laboratory to exclude the possibility of a high-dose ‘hook’ effect [10,11]. The Alpha CrAg enzyme-linked immunosorbent assay (EIA) (Immuno-Mycologics) was also performed in duplicate on referred specimens. India ink staining of the CSF was performed by the referring laboratories and repeated by the reference laboratory on available specimen (cultured isolate suspended in saline for case 1, CSF and cultured isolate for case 2). Phenotypic biotyping was performed at the reference laboratory by culturing clinical isolates on different media, either Staib’s niger-seed medium or L-canavanine glycine bromothymol blue (CGB) agar [12,13]. Subsequently, isolates were routinely cultured on Sabouraud dextrose agar for 48 h at 30 °C. Confirmation of identification of the cultured isolates was based on both matrix-assisted laser desorption ionization-time of flight mass spectrometry (MALDI-TOF) (Bruker Biotyper, Billerica, United States) and sequencing of the internal transcribed spacer (ITS) region and large subunit (LSU) of the ribosomal gene [14]. Genotypic characterisation of cultured isolates was based on the seven consensus loci for multilocus sequence typing (these loci include the housekeeping genes; *CAP59*, *GPD1*, *LAC1*, *PLB1*, *SOD1*, *URA5* and the IGS1 region) [15]. Cultured isolates were also examined ultrastructurally using transmission electron microscopy (TEM) with a modified protocol initially developed for bacterial capsular studies [16]. Briefly, capsular polysaccharides were stabilized and stained with 0.075 M l-lysine acetate (Fluka, Sigma-Aldrich, Darmstadt, Germany, CAS Number 57282-49-2) and 0.075% ruthenium red (Sigma-Aldrich, CAS Number 11103-72-3), in a 20 min prefixation, with 5% glutaraldehyde and 6.25% methanol-free formaldehyde in 0.1 M sodium cacodylate buffer, pH 6.8. Cells were then fixed for a further 5 h without L lysine acetate, rinsed with chilled ruthenium red-containing buffer, post-fixed in 1% osmium tetroxide for 90 min, buffer rinsed, gradually dehydrated in a graded ethanol series, and infiltrated with and embedded in LR white resin (London Resin company, Agar Scientific, United Kingdom). All stages were performed on ice, and all reagents contained 0.075% ruthenium red, with the exception of the two final dehydration stages (90% ethanol, 100% ethanol). Comparisons were made between the cells of the clinical isolates and those of an American type collection (ATCC 34875) control culture grown under the same conditions and processed simultaneously. Capsule measurements were made of medially-sectioned cells using calibrated software (OSIS) on an FEI BioTwin Spirit TEM fitted with an Olympus Quemesa CCD camera. Statistical analyses were performed using the Kruskal-Wallis H test for non-parametric data.

Surveillance of essential communicable diseases and the publication thereof, is carried out at the NICD with the approval of the Human Research Ethics Committee (Medical) of the University of the Witwatersrand, ethics protocol number M160667. Written consent from the attending clinicians was obtained prior to the publication of the anonymised case reports below (both patients deceased).

## 3. Results

### 3.1. Case 1

A 36-year-old male was admitted to a general hospital in Gauteng Province in May 2017 for investigation of acute psychosis. Laboratory investigations on admission (day 1): Negative serology for *Treponema pallidum*, values in the normal range for thyroid-stimulating hormone, full blood cell count and vitamin B12, elevated serum levels of C-reactive protein, alkaline phosphatase and gamma-glutamyl transferase, with low serum albumin levels and hyponatraemia (Table 1). Further investigations on day 3 included a lumbar puncture (LP), with normal CSF chemistry, 20 erythrocytes/µL and cell counts of zero for polymorphonucleocytes and lymphocytes. Both CrAg LFA (in duplicate) and India ink staining of the CSF were negative, although on the CSF culture there was heavy growth of *C. neoformans*. The patient had a CD4 count of 19 cells/µL, which prompted reflex screening for cryptococcal antigenaemia. Again, the CrAg LFA was negative. The CSF specimen and the clinical isolate were referred to NICD for further testing.

**Figure 1:**
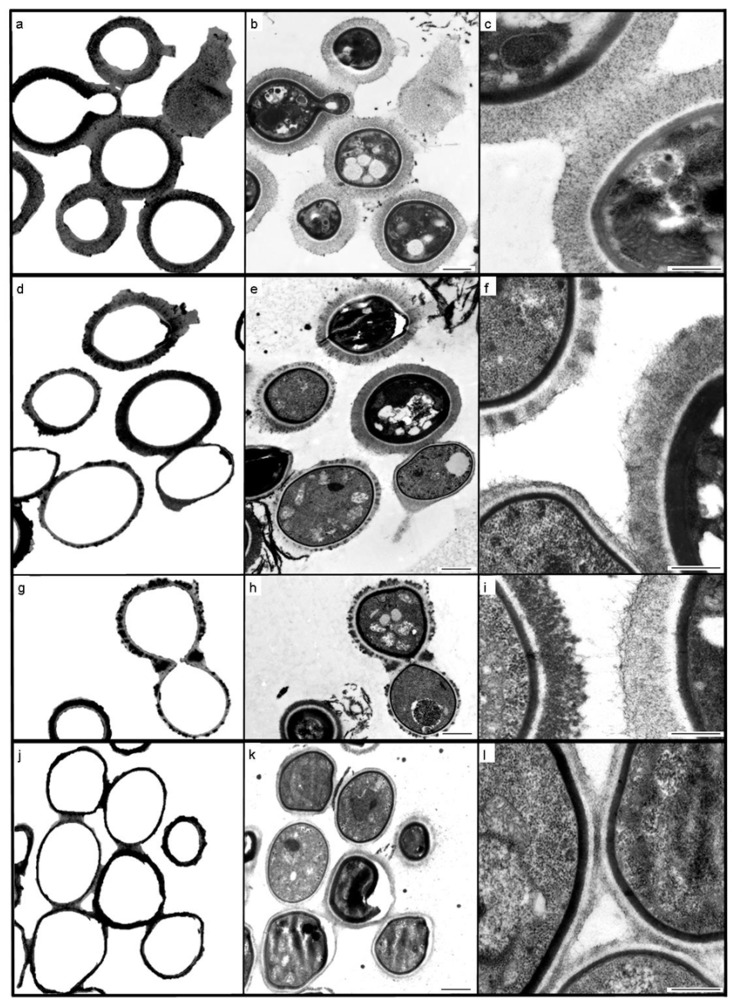
Transmission electron microscopy of cultured *Cryptococcus neoformans* cells: American Type Collection (ATCC) 34875 (**a**–**c**); case 1 isolate (**d**–**i**); case 2 isolate (**j**–**l**); where (**a**,**d**,**g**,**j**) (internal structure digitally subtracted) are paired with (**b**,**e**,**h**,**k**) (complete original images) in order to highlight capsule thickness variation. Figures (**c**,**f**,**i**,**l**) illustrate the homogeneous, delicately fibrous, regular capsule of the ATCC control (**c**); the variably osmiophilic, peripherally matted or loosely fibrous capsules of isolate 1 (**f**,**i**); and the thin, tomentose capsules of isolate 2 (**l**). Scale bars = 1 µm (**b**,**e**,**h**,**k**) and 0.5 µm (**c**,**f**,**i**,**l**).

**Figure 2:**
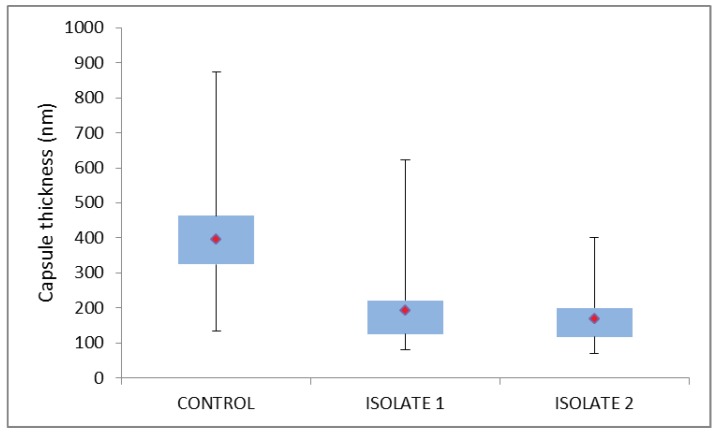
Comparison of capsule dimensions measured from median sections through cultured cells (*n* = 100), with the mean capsule thickness for each culture indicated by the red diamond within the box (2nd and 3rd quartiles), with the whiskers indicating the entire range of measured capsule thickness.

At NICD, possible pre-analytical errors (such as specimen collection from the wrong patient, or mislabeling) and analytical errors (such as specimen mix-up, incorrect specimen processing, a haemolysed specimen, incorrect reading time for the CrAg LFA, poor visual acuity of laboratory personnel, incorrect interpretation of CrAg LFA results, transcription errors) were excluded after thorough investigation. The CrAg LFA continued to yield a negative result despite the use of kits from three different lots, and serial dilution testing (titration 1:1280) to exclude ‘hook’ effects. The CrAg EIA yielded an optical density of 0.630/0.778 (positive result) on the CSF specimen. The identification of *C. neoformans* was confirmed phenotypically with the development of brown-pigmented colonies on Staib’s niger-seed medium, and no colour change (negative) growth on CGB agar. *C. neoformans* was confirmed by MALDI-TOF. Sequencing of the ITS region and LSU of the ribosomal gene also confirmed that this isolate was *C. neoformans*, whilst MLST revealed that this isolate had the molecular type VNI (serotype A) and sequence type (ST) 31. Insufficient residual CSF meant that the reference laboratory performed India ink staining on the cultured isolate (cells suspended in saline for staining), but still failed to detect any encapsulated cells. Ultrastructural comparison of the cells of the clinical isolate, with those of the control culture (ATCC 34875) illustrated obvious differences between the two cultures that could be quantified, and which were shown to be statistically significant when compared with the control cells (*p* = 0.001) (Figure 1 and Figure 2). The extensive and uniformly reticulate nature of the capsule of control cells contrasted markedly with that of the case 1 isolate, which was notable for its variability in texture, appearance and thickness. Case 1 capsule was seen to be reduced in comparison with the control isolate (mean of 192 nm compared with 395 nm in the control; *n* = 100).

On diagnosis of CM, the patient received amphotericin B deoxycholate-based induction treatment, but continued to deteriorate clinically and subsequently died one week later.

### 3.2. Case 2

A 51-year-old female was admitted to a hospital in the Eastern Cape Province in June 2017, for investigation of seizures. Laboratory investigations on admission (day 1) revealed a non-reactive rapid plasma reagin test, no hepatitis B virus surface antigen, a CD4 count of 119 cells/µL, hyponatraemia, low serum chloride concentrations, borderline abnormal liver function with elevated serum alkaline phosphatase and gamma-glutamyl transferase (Table 1), and peripheral white blood cell counts and haemoglobin within normal ranges (4.5 to 11.0 × 10^9^/L and 12.0 to 15.5 g/dL in women), but with a lowered platelet count (121 × 10^3^ cells/µL; normal range 150–400 × 10^3^ cells/µL). CSF parameters from the initial, and subsequent, lumbar puncture(s) are tabulated (Table 1). A CSF CrAg LFA and latex agglutination test, and CSF India ink staining were negative, although on culture of CSF, there was a heavy growth of *C. neoformans*—an identification confirmed by VITEK mass spectrometry (bioMeriéux, Marcy-l’Étoile, France). Once again, the CSF specimen and clinical isolate were referred to NICD for further testing.

The phenotypic identification of *C. neoformans* made by the referring laboratory, was confirmed at the NICD by culture (Niger seed agar positive, CGB agar negative), which was supported by Bruker Biotyper MALDI-TOF results, molecular identification (*C. neoformans*) and genotypic characterisation (molecular type VNI [serotype A] and ST31). However, repeat India ink staining was negative (CSF and cultured cells), as were 4 different kit lots for CrAg LFA, a 1:5 to 1:1280 titration series with CrAg LFA, and a negative CrAg EIA on CSF (optical density −0.034/−0.039). The clinical isolate was also referred for capsule characterisation using TEM, which revealed the presence of a consistently thin capsule (mean of 168 nm; *n* = 100), with a uniformly fibrous, tomentose appearance, and very much reduced in comparison with that of the ATCC control cells (Figure 1 and Figure 2). Once again, the measured difference between the capsule thickness of control cells and those of the case isolate, were statistically significant (*p* = 0.0001).

On diagnosis of CM, the patient received standard treatment with amphotericin B deoxycholate + fluconazole (1200 mg daily for two weeks) and completed the induction phase of treatment uneventfully. CSF taken on day 6 proved to have no cryptococcal growth on culture (14 days culture incubation). By day 23, she was clinically well and was discharged.

The patient was readmitted on day 43. Few details are available other than an LP report (Table 1) and a laboratory test result which was a positive blood culture for extended spectrum beta-lactamase-producing *Escherichia coli*. The patient died shortly after admission.

## 4. Discussion

In these two cases, the initial laboratory investigations were negative for cryptococcosis by a CSF CrAg LFA and India ink staining, although *C. neoformans* was identified by culture of CSF, and by MALDI-TOF, in both cases. There are two possibilities for the failure of repeated CrAg LFAs in providing a positive result in the presence of culture-confirmed cryptococcal meningitis: Either a serotypic bias in the assay antibodies, or the level of antigen in the specimens being so reduced as to be undetectable by this assay. Serotypic bias is unlikely, given that both cases were caused by VNI (serotype A), in which capsular polysaccharides are intermediate in the extent of both xylose substitution and O acetylation on the mannan backbone (as monoclonal antibodies react poorly when there is the least O acetylation and the greatest xylose substitution, as in serotype C) [17].

The fact that both clinical isolates had TEM-discernible capsules suggests that these capsular variants had insufficient polysaccharide for detection by the CrAg LFA. The TEM results of the two clinical isolates suggest that cryptococcal disease with cells having a mean capsular thickness ≤192 nm (as in case 1) is below the threshold of detection for this assay. The observation of hypocapsular phenotypes may be linked to the rapid clearance by day 6 in case 2, as findings on murine cryptococcosis indicate that ‘acapsular’ variants are more rapidly cleared [18].

There are a number of reports of capsule-deficient and acapsular isolates based on light microscopy and India ink staining—most recently that by Mahajan et al. [19]. Environmental variables are known to influence capsule expression, in that cryptococcal strains exhibit physiological differences in vitro and in vivo, the polysaccharide capsule gets thicker with age and the changing environmental conditions associated with mammalian cell infection, and the capsule is much larger in vivo than under normal in vitro conditions [18,20,21,22]. TEM, with conventional specimen processing protocols, was used to investigate an unusual case of pulmonary cryptococcosis [23]. To our knowledge, TEM with a cationic probe for capsule staining has not been applied to cryptococcal capsular characterisation previously.

In a recent publication based on clinical isolates obtained from HIV/AIDS patients in Botswana [21], the mean capsule thickness of *C. neoformans* VNI isolates was 5.5 µm ± 2.7 µm. The difference in mean capsule thickness between this study and the cells of our study is partly due to the fact that as in almost all previous publications describing capsule thickness as a virulence factor, the capsule of cells was negatively stained with India ink and measured using light microscopy, which has a 200 nm limit of resolution (thus the two case isolates would have been described as acapsular, and indeed, no capsule was evident using this technique when performed by both the referral and reference laboratories). Although an electron microscope has resolving power orders of magnitude greater than this thereby enabling accurate measurement down to single nanometers, the measured dimensions may be under-estimated given that the TEM preparative protocols for biological material may cause shrinkage due to chemical fixation and denaturation, with possible loss of some capsular material, which cannot be stabilised and stained completely. However, the growth of the two clinical isolates and the control culture under the same conditions for the same length of time, with simultaneous processing for TEM, permits at least a relative capsular comparison.

It also raises the question of whether or not any truly acapsular variants could be described when using TEM with a capsular staining protocol, particularly as the CAP59 sequences obtained from MLST showed no variation for these two cases. However, nucleotide differences in the MLST sequences of CAP59 (which codes for capsular associated protein) may only reveal new allele types and may not be necessarily related to the capsular phenotype, and although acapsular phenotypes have been associated with mutations in other genes such as CAP10, CAP60 and CAP64, the mutant cells still secreted glucuronoxylomannan-like polymers and galactoxylomannan [24]. It would be interesting to determine whether or not these capsular polysaccharides could be visualised using the TEM protocol utilised in the two cases reported here, and whether or not conformational changes in the capsular components are reflected ultrastructurally. This future research would entail concurrent investigations on the effect on the capsule of varying environmental culturing conditions, with extensive sequencing (at least of all known genes involved in capsule production, formation and expression), light and electron microscopy, and biochemical analyses. Based on the findings from this research, it would also be of value to investigate various capsular characteristics in relation to the immune responses of hosts. A multidisciplinary approach to these cryptococcal issues is essential.

## Figures and Tables

**Table 1:** Laboratory test results for cases 1 and 2.

Laboratory Testing	Case 1	Case 2				Normal Range
CSF PARAMETER	Day 3	Day 1	Day 6	Day 13	Day 43	
Chloride (mmol/L)	111	96	104	127	129	118–132
Glucose(mmol/L)	3.6	0.1	1.3	2	2.6	2.5–4.4
Protein (g/L)	0.22	3.57	2.56	0.88	0.65	0.15–0.45
Adenosine deaminase (U/L)	*	10.7	17.9	9.2	0	<6
Polymorphonucleocytes (cells/µL)	0	2	4	0	0	<5
Lymphocytes (cells/µL)	0	163	463	647	6	<5
Erythrocytes (cells/µL)	20	19	0	>10,000	125	0
India ink	Negative	Negative	Negative	Negative	Negative	Negative
CrAg LFA **	Negative	Negative	Negative	Negative	Negative	Negative
Culture	*C. neoformans*	*C. neoformans*	No growth	No growth	No growth	No growth
SERUM CHEMISTRY	Day 1	Day 1	Day 6	Day 13	Day 43	
Sodium (mmol/L)	126	*	125	129	*	136–145
Potassium (mmol/L)	3.3	*	3.8	haemolysed	*	3.5–5.1
Urea	4.4	*	11	9.5	*	2.1–7.1
Creatinine (µmol/L)	70	*	124	388	*	64–104
C–reactive protein (mg/L)	92	*	*	336	*	<10
Total protein (g/L)	82	*	*	55	*	60–78
Albumin (g/L)	22	*	*	16	*	35–52
Total bilirubin (µmol/L)	16	*	*	58	*	5–21
Conjugated bilirubin (µmol/L)	5	*	*	40	*	0–3
Alanine transaminase (U/)L	36	*	*	38	*	7–35
Aspartate transaminase (U/L)	51	*	*	181	*	13–35
Alkaline phosphatase (U/L)	380	*	*	237	*	42–98
Gamma–glutamyl transferase (U/L)	279	*	*	218	*	<40

* Information not available. ** Cryptococcal antigen lateral flow assay.
